# Eczema, acne, and psoriasis in people with skin of colour: a qualitative UK-based study

**DOI:** 10.3399/BJGP.2025.0720

**Published:** 2026-05-19

**Authors:** Eliza J Hutchison, Roxanne M Parslow, Hannah E Wainman, Matthew J Ridd

**Affiliations:** 1 Centre for Applied Excellence in Skin and Allergy Research, University of Bristol, Bristol, UK; 2 Dermatology Department, Royal United Hospitals Bath NHS Foundation Trust, Bath, UK; 3 Dermatology Department, University Hospitals Bristol and Weston NHS Foundation Trust, Bristol, UK

**Keywords:** dermatology, diversity, primary health care, qualitative resarch, skin of colour

## Abstract

**Background:**

Inflammatory dermatoses have a varied prevalence and appearance in diverse skin tones. The underrepresentation of people with skin of colour in medical education and clinical trials is widely acknowledged. However, there has been limited research on experiences from a patient perspective.

**Aim:**

To explore the experiences of eczema, acne, and psoriasis in adults with skin of colour in the UK.

**Design and setting:**

This was a qualitative study of 20 people in the UK with eczema, acne, and psoriasis and skin of colour, recruited using online methods.

**Method:**

Participants took part in online, one-to-one, semi-structured interviews. NVivo qualitative data analysis software was used to code and organise the data. Reflexive thematic analysis was used to generate themes using an iterative approach.

**Results:**

Participants were mostly female (65%, 13/20), Asian/Asian British ethnicity (45%, 9/20), and had eczema (55%, 11/20). We identified eight themes: delayed or missed diagnosis; preferences regarding healthcare professionals; lack of online information and social media use; misunderstanding in cultural communities; concerns about treatment and lack of research in skin of colour; complementary medicine use; experiences and impact of dyspigmentation; and challenges with structural racism.

**Conclusion:**

The themes generated highlight the unique experiences and challenges faced by UK adults with eczema, acne, and psoriasis. The findings can help guide diagnostic approaches, culturally sensitive communication, and treatment discussions for patients with skin of colour. Further research is needed in this underrepresented group.

## How this fits in

This study is the first, to the authors’ knowledge, to explore the experiences of living with an inflammatory dermatosis specifically in people with skin of colour. We generated eight themes important to participants: delayed or missed diagnosis; preferences regarding healthcare professionals; lack of online information and social media use; misunderstandings in cultural communities; concerns about treatment and lack of research; complementary medicine use; experiences and impact of dyspigmentation; and challenges with structural racism. These findings offer insight into the complex experiences and challenges faced by UK adults with skin of colour living with eczema, acne, and psoriasis. Our practical points for primary care clinicians are a step towards facilitating mutual understanding and improving care for people with skin of colour.

## Introduction

Skin problems are the most frequent reason for primary care consultation in the UK,^1^ discussed in 14.2% of all adult appointments.^2^ The majority of these patients are managed solely in primary care.^2^ People with skin of colour represent a diverse population of ethnic groups, including but not limited to those of an African, Asian, and Middle-Eastern descent.^3^ The prevalence and appearance of common inflammatory dermatoses, such as atopic dermatitis/eczema, acne, and psoriasis vary across different ethnicities and skin types.

Eczema has a UK prevalence of 2.6%,^4^ with higher rates in people of Asian, Black, and Mixed ethnicities.^5^ Features such as extensor involvement and follicular and discoid lesions are more prominent in darker skin.^6^ Psoriasis has a UK prevalence of 2.8% with a lower incidence among individuals with skin of colour,^7^ although this may be underestimated because of their underrepresentation in research.^8^ Black people may have thicker, scalier plaques and Asian people can have more severe disease.^9^ Acne affects 85% of teenagers and can persist into adulthood, affecting 12% of adult women.^10,11^ It has a higher incidence in females of African American, Hispanic, and Asian descent.^12^ Asian people may have more inflammatory lesions and pomade acne is more common in Black people secondary to comedogenic hair products.^12^ In all inflammatory dermatoses, the redness typically seen in white skin is less conspicuous in skin of colour and instead lesions may appear brown, grey, or violaceous.^13^ Skin of colour also carries a greater risk of post-inflammatory hyperpigmentation that can be associated with significant psychological distress.^12^

In recent years the number of publications relating to skin of colour has rapidly expanded.^14^ Research to date focuses on topics such as underrepresentation in medical curriculae,^15,16^ educational materials,^16,17^ and clinical trials.^18^ There is little research exploring experiences with skin of colour from a patient perspective. The aim of this study was to explore the experiences of eczema, acne, and psoriasis in adults with skin of colour in the UK with a primary care focus.

## Method

### Study design

First, we conducted an online cross-sectional survey (25 February to 1 July 2025) that was open to individuals of all skin tones with eczema, acne, and psoriasis, the results of which will be published separately. Eligible participants with skin of colour were purposively sampled from the survey responses to take part in the qualitative interviews. One-to-one interviews were semi-structured to enable rich data collection on this complex, multifaceted subject.

### Participant recruitment

Participants had to be >16 years; living in the UK; have a diagnosis of eczema, acne, or psoriasis; and able to speak English. Online recruitment methods were used to ensure representation of diverse experiences from across the UK. The study was advertised on social networking platforms, websites, and mailing lists with the assistance of skin charities, research organisations, social media groups, and online influencers. Based on responses to the online survey, individuals with skin of colour who consented to be contacted were sent an email invitation to participate in an online interview. Purposive sampling was used to recruit individuals with a varied representation of age, diagnosis, and self-identified gender and ethnicity. A sample size of 20 participants was estimated to achieve thematic saturation and gain adequate information power such that no new insights were identified by the time of completion.^19,20^ We offered interview participants a £25 reimbursement via bank transfer or voucher for taking part in the interview.

### Data collection

A topic guide was developed and piloted with patient representatives to ensure it covered the most salient topics for prospective participants. It was structured using open questions with prompts to promote richer data collection. Semi-structured, one-to-one interviews were conducted from 18 March to 17 June 2025. All interviews were conducted virtually by the first author using Microsoft Teams. The topic guide was flexible and evolved as the interviews progressed and themes were generated (Supplementary Information S1). For example, additional questions were incorporated to explore experiences of stigma and research concerns. Interviews were audio-recorded and automatically transcribed verbatim using the inbuilt Microsoft Teams function. Following the interviews, transcriptions were checked for accuracy and anonymised by the first author.

### Data analysis

NVivo (Version 14) qualitative data analysis software was used to code the data, organise data into higher-level themes, and provide an audit trail of the analysis. Braun and Clarke’s reflexive thematic analysis method was used to analyse qualitative data.^21^ This is a flexible qualitative analytic method used to generate themes through active engagement with the data.^21^ It aligns with the constructivist paradigm by acknowledging that reality is subjective, socially constructed, and shaped by individual perspectives.^22^ This is an appropriate method of data analysis to identify patterns of meaning from interviews and generate wide-reaching codes to represent personal, complex experiences.

The first author analysed all interview transcripts in line with Braun and Clarke’s six-step process.^23^ A hybrid approach of both deductive and inductive approaches to analysis was used.^24^ Initial coding was deductive and used the interview topic guide as a framework. Examples of initial codes generated include: colour change; covering up; community stigma; feeling dismissed; and natural products. Subsequently an inductive approach was adopted to help develop and refine themes grounded in the data. Meetings between investigators took place to discuss emerging themes and reach a consensus regarding final themes. An iterative approach was taken, repeating cycles of data collection and analysis until thematic saturation was achieved and no new themes were generated.

### Reflexivity

Researcher reflexivity was adopted throughout the data collection and analysis stages.^25^ The first author conducted all interviews and is a practising dermatologist who identifies as a White British female. The first author’s clinical role was not disclosed unless directly enquired about by participants, based on evidence that disclosing an interviewer’s clinical role may lead to perceived power imbalances and selective information sharing in qualitative studies.^26^ The potential impact of differing ethnicity between interviewer and interviewee on data collection and analysis was also acknowledged. Reflexive field notes, taken after the interviews, were used to develop themes and enhance the transparency of the analysis. Senior researchers, the senior author (White male, GP) and the second author (White female with experience in qualitative research and eczema) independently reviewed and double coded 10% of the interview transcripts to help reach a consensus on the final coding and ensure a diversity of perspectives when interpreting the data.

### Patient and public involvement

Patients and members of the public with a lived experience of the study topic were actively involved in the design, conduct, and dissemination of this study. Feedback was obtained in the form of online meetings and email communication at regular intervals throughout the study. Their feedback directly informed key study elements including the recruitment advert and topic guide.

## Results

The survey had 135 responders, of which 73 identified as being of non-White ethnicity. Of these eligible participants, 54 indicated that they would like to take part in an online interview and were all contacted via email (Table 1). Of those contacted, 46 responded via email confirming an interest; three of these subsequently did not respond, and one booked but did not attend the interview. Of the remaining 42 individuals, a total of 20 one-to-one interviews were conducted based on purposive sampling to reach data saturation (Table 1).

**Table 1.** Characteristics of eligible survey responders contacted and interview participants

Characteristics	Eligible survey responders contacted, *n* (%)	Interview participants, *n* (%)
**Total**	54	20
**Age, years**		
16–24	15 (28)	4 (20)
25–34	24 (44)	7 (35)
35–44	6 (11)	5 (25)
45–54	2 (4)	1 (5)
55–64	5 (9)	1 (5)
≥65	2 (4)	2 (10)
**Gender**		
Female	35 (65)	13 (65)
Male	18 (33)	7 (35)
Non-binary	1 (2)	0 (0)
**Ethnicity**		
Asian or Asian British	22 (41)	9 (45)
Bangladeshi	4	1
Indian	12	5
Malaysian	1	1
Pakistani	2	1
Taiwanese	1	1
Other	2	0
Black, Black British, African, Caribbean	27 (50)	7 (35)
African	21	5
Black British	1	0
Caribbean	5	2
Mixed or multiple ethnic groups	5 (9)	4 (20)
British, Indian, and Portuguese	1	1
Malaysian, Chinese, and Japanese	1	1
White and Asian	1	1
White and Black Caribbean	1	1
White and North African Arab	1	0
**Diagnosis**		
Acne	10 (19)	3 (15)
Eczema (atopic dermatitis)	28 (52)	11 (55)
Psoriasis	12 (22)	6 (30)
Combination of above	4 (7)	0 (0)
**Dermatology Life Quality Index**		
0–1 (no effect at all on patient’s life)	1 (2)	1 (5)
2–5 (small effect)	13 (24)	5 (25)
6–10 (moderate effect)	12 (22)	3 (15)
11–20 (very large effect)	15 (28)	4 (20)
21–30 (extremely large effect)	13 (24)	7 (35)
**Region**		
England	43 (80)	16 (80)
East Midlands	2	1
London	16	7
North East	1	0
North West	5	1
South East	6	4
South West	11	3
West Midlands	2	0
Scotland	8 (15)	1 (5)
Wales	3 (6)	3 (15)
**Mean-tested benefits or earns less than national living wage**		
No	33 (61)	13 (65)
Yes	18 (33)	7 (35)
Prefer not to say	3 (6)	0 (0)

Interview participants were mostly female (65%, 13/20), Asian or Asian British ethnicity (45%, 9/20), and had eczema (55%, 11/20). Ages ranged from 22 to 66 years (mean 36 years, standard deviation 14 years). Dermatology Life Quality Index score ranged from 1 to 28 (out of 30) with a mean score of 13.7 (standard deviation 9.4), indicating a ‘very large effect’ on a patient’s quality of life. A total of seven of the 20 (35%) participants received means-tested benefits or earnt less than the national living wage, the majority of whom were of a Black African ethnicity.

Interview duration ranged from 17 to 60 min (mean 34 min, standard deviation 11 min). Eight key themes were generated, demonstrated in Figure 1. Representative participant quotes with identifiers are provided for each theme, with additional pertinent examples incorporated into the main text.

**Figure 1.**
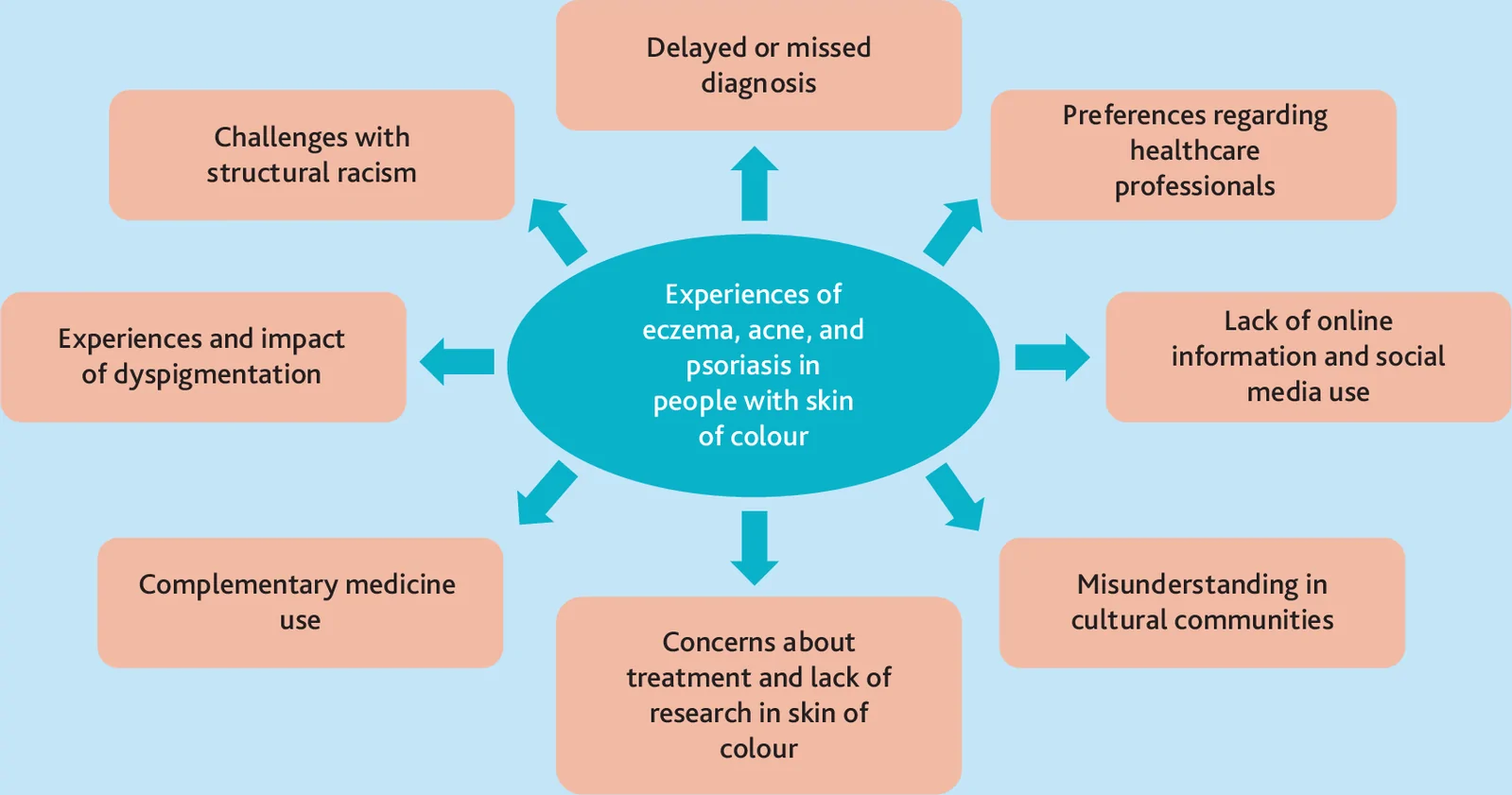
Themes generated from interviews.

### Delayed or missed diagnosis

Experiences regarding diagnosis were varied, with some experiencing a delayed or misdiagnosis of their skin condition whereas for others it was more straightforward. One participant recalled their GP *‘straight away looked at it and said, that’s psoriasis’*. In contrast, some waited years for a diagnosis and several participants were initially dismissed as having a *‘hygiene issue’* or misdiagnosed with a *‘fungal infection’*, or *‘scabies’*:

*‘The GP just had no idea and there were two of them looking at it and they were like, “oh maybe it’s scabies”. And it’s like, it’s not scabies. It’s just really basic stuff that they weren’t able to identify because they haven’t seen anything like it. It’s a really White practice.’* (P9, 57 years, Asian female, psoriasis)

Some attributed this to their skin colour and ethnic background, with one participant saying:

*‘When a Black patient walks in with purple plaques and grey patches,* [healthcare professionals] *don’t see it as that’s the problem, they just dismiss it as a hygiene issue.’* (P20, 29 years, Black male, psoriasis)

Some voluntarily highlighted that they were misdiagnosed by clinicians from their own ethnic background. Several individuals highlighted the distinctive changes in their skin tone compared with white skin, particularly the lack of redness, which *‘didn’t look as bad’* and they *‘didn’t associate’* it with an inflammatory skin condition:

*‘The appearance of eczema on my tone of skin, sometimes it doesn’t look what you think looks like eczema, if that makes sense. So it’s more dark, my skin appears very, very dark. And I can’t see any redness in my skin tone really.’* (P3, 43 years, Asian female, eczema)

Some felt they ‘got lucky’ with their GP when they were diagnosed without delay and said *‘more training’* in skin of colour is needed for clinicians.

### Preferences regarding healthcare professionals

Participants’ preferences varied regarding characteristics of the clinician they consult with. Some strongly felt there was a *‘disconnect’* with White clinicians and preferred someone of the same ethnicity. One Black female strongly felt that with a Black female clinician there would be a better sense of *‘empathy’* and *‘rapport’*:

*‘I was hoping that my dermatologist would be Black and would be female because I think she would really understand me, even in terms of culture and how things are. But then when I saw it was a White doctor … I felt like there was a bit of a barrier there.’* (P8, 23 years, Black female, eczema)

However, some perceived clinicians of the same ethnicity as being *‘even more dismissive’*. Many felt it did not matter and instead prioritised clinicians’ knowledge and communication skills such as *‘listening’* and *‘being empathetic’*:

*‘To me, it doesn’t matter about colour or race or anything as long as you have the knowledge in the field that you’re speaking about.’* (P14, 26 years, Mixed ethnicity female, eczema)

Preferences regarding professional roles varied, with positive and negative experiences of consulting dermatologists, GPs, and allied health professionals. Again, some felt the role was less relevant, and it was more important to *‘know what you’re doing’*:

*‘I don’t think that it matters. Because when you go in, the main goal is just to get* [your skin] *under control … I don’t think there’s any difference between the nurse, the associates, the doctors.’* (P10, 31 years, Asian female, eczema)

### Lack of online information and social media use

Some individuals commented that representation of skin of colour in online resources such as the NHS website had *‘improved’*, but most felt it remains underrepresented and *‘a lot more is definitely needed’*. Several highlighted the predominance of *‘red rashes’* that meant they found it difficult to recognise their skin condition because *‘it didn’t look like psoriasis’*. One participant stated:

*‘I feel like it’s always the secondary part. In the NHS it will be like this is the skin stuff for white skin, and then in darker skin tones it may appear grey or black. It’ll be like that as a sentence. I think having a dedicated page on eczema in darker skin or people with colour would help because then you could go into detail about things like hyperpigmentation.’* (P6, 27 years, Asian male, eczema)

Several participants, predominantly young females, sought information about their skin condition on social media platforms such as Instagram and TikTok, and follow social media influencers with skin of colour because they are more relatable. One female of Mixed ethnicity said you can *‘find things out quicker as a person of colour’* on social media platforms compared with NHS resources. For some, social media use was a *‘freeing’* experience that made them feel *‘confident’* about their skin and helped them understand issues such as hyperpigmentation:

*‘There’s this woman* [online] *called Eczema by Harriet. And she’s a Black woman and she has eczema and topical steroid withdrawal. And seeing her page … I was like, oh my God, me and Harriet have the same skin, my skin looks like Harriet’s. And it felt so freeing because I’ve never really had that in all my life. Even with the GPs and all the leaflets that I see about eczema, you hardly see black skin.’* (P8, 23 years, Black female, eczema)

*‘There’s a good community I find online with excellent stuff like that. So it’s nice to see people with skin of colour and their experiences of their skin either darkening or lightening or just general discolouration across our whole body. It’s nice to know that it’s not a single experience basically.’* (P16, 27 years, Mixed ethnicity female, eczema)

One Black female said it made her feel *‘happy’* to see people *‘whose skin looks exactly like mine’* because many people in her culture *‘hide it’*. However, some discussed the unreliability of social media as information is *‘not backed up scientifically’*, and one individual described Facebook groups as *‘garbage’*.

### Misunderstanding in cultural communities

Participants of all ethnicities highlighted *‘superstition’* and *‘misinformation’* in their cultural communities, where there remains significant misunderstanding surrounding the cause and treatment of inflammatory dermatoses. Conditions such as eczema are considered *‘something a bit dirty rather than a genuine medical condition’* and there is *‘confusion and misinformation about how to actually treat it’*. One participant said:

*‘Especially where I come from, there’s this understanding that eczema is a communicable disease. If I put a shirt on and the same shirt somebody else puts on, you might likely get infected with eczema so there’s that stigmatisation around me … we are quite scared.’* (P19, 66 years, Black male, eczema)

Another participant was told by his relative that *‘eczema is a White person thing’* so he felt *‘broken’*.

Many said *‘stigma’* and *‘taboo’* still surround skin conditions in their communities. One participant with eczema stated:

*‘I don’t know whether people of my community and my skin tone are as open when it comes to eczema. So even if they do have eczema, it’s more kind of hidden away and shut up about and not openly talked about so they don’t have these conversations with healthcare professionals.’* (P3, 43 years, Asian female, eczema)

One Black participant raised that there were issues which intensified the challenges of having psoriasis:

*‘In the Black community, they talk about strength and it’s sacred and you always want to hide illness. And your skin is actually tied to your pride. They talk about melanin skin and everybody wants it to be so perfect, and yours isn’t as perfect as it’s supposed to be.’* (P20, 29 years, Black male, psoriasis)

Many experienced negative comments and pressure from family and community members, with several females facing concerns about marriage prospects because of their condition. It was suggested that community initiatives led by people with skin of colour could help tackle cultural misunderstandings and encourage dialogue.

### Concerns about treatments and lack of research in skin of colour

Several participants were concerned about treatment efficacy in their skin type. One participant was concerned about seeing an image of *‘a White person’* on the medication packaging that dissuaded him from using it because *‘we don’t have the same skin tone’*:

*‘It’s kind of different because I feel the Western medicines are not really effective on a Black body because of the way genes are different.’* (P20, 29 years, Black male, psoriasis)

One Black participant with acne was specifically concerned about a topical treatment having a *‘bleaching’* effect and causing skin lightening:

*‘With the cream I was using, it had quite a low percentage of bleach or peroxide kind of thing. So I always thought if I was to do anything more, or get something better or stronger, it’ll just bleach my skin. And I’ve seen how people from my culture, they use bleaching creams and stuff, and I’ve seen how bad it can be … I’d rather just live with* [acne] *than put myself through that.’* (P13, 46 years, Black male, acne)

A lack of research on people with skin of colour seemed to contribute to doubts around treatment efficacy, with fears of being *‘a guinea pig’* using treatment that has not been researched in skin of colour:

*‘When the statistics and all the backup information suggests that it’s not even been tested on people of my skin colour why should I not question the adversity of what’s going to happen next because of taking that medication … you don’t want to be a guinea pig.’* (P2, 41 years, Asian female, psoriasis)

These same concerns were also a suggested reason why people from diverse ethnic backgrounds are reluctant to participate in research, with one individual saying he felt *‘embarrassed’* to ask people to participate in this study.

### Complementary medicine use

Many reported using complementary treatments for their skin condition. Frequently used complementary treatments included oils and herbal remedies, used across all ethnic groups, and homeopathy and Ayurvedic medicine that were more prevalent among Asian individuals.

Use was often culturally driven, with one individual explaining that for skin conditions:

*‘There’s more of a thing in our culture to be treated by natural remedies rather than medicines. Whereas if you have a heart condition, people are like, “oh take your medicine, there’s nothing natural that will cure it”. I think it’s just because it’s on the skin.’* (P1, 22 years, Asian female, eczema)

In many cases, complementary medicine use was *‘passed down from generations’* and driven by parental preferences during childhood, as exemplified in these two quotes:

*‘*[My mum] *was thinking it was something from inside probably like bacteria, so she tried local herbs to actually get in there to kill it from the inside ... I think this thing was passed down from generations from her mum and her grandparents as well, so it’s something that always sticks in the family. So I think she was OK with the traditional because she feels it’s better than the Western medicines.’* (P20, 29 years, Black male, psoriasis)

*‘Before I went to see a dermatologist, my mum took me to see a homeopathic practitioner of medicine. And I was quite sick actually, my skin was in a real mess. And she gave me some garbage homeopathic thing to try and obviously it didn’t work.’* (P11, 22 years, Asian male, eczema)

One individual said his parents were *‘pushed a little bit by the community to go down that route’*. Other driving factors included a desire to avoid *‘medication from White people’*, greater confidence in products commonly used in people’s country of heritage because it’s *‘something we already do’*, and a stronger sense of *‘connection’* with ethnically concordant complementary practitioners.

For others, use had less of a cultural association and instead was driven by *‘frustration’* that prescribed treatments were not working and a fear of side effects, particularly regarding topical steroids and immunosuppressants.

Despite the widespread use of complementary medicine, some described a negative experience and termed it *‘garbage medicine’*, often stopping once they reached adulthood.

### Experiences and impact of dyspigmentation

One of the most frequently discussed themes throughout all interviews was dyspigmentation. All participants reported hyperpigmentation, with some also experiencing hypopigmentation. Several individuals likened being multi-tonal to animals such as a *‘dalmatian’*, *‘leopard’*, or *‘raccoon’*. Colour change was mostly attributed to skin inflammation, but some felt it was secondary to topical treatments.

Dyspigmentation had a significant impact on participants of all genders, ages, and ethnicities. Many were *‘self-conscious’* and covered it using make-up or clothing, with one individual explaining that Black people have *‘a lot of pressure to look a certain way’*. Some received negative comments from within their own community, such as being told *‘your skin looks dirty’*. Dyspigmentation also had a profound psychological impact with sequelae such as *‘body dysmorphia’* and social isolation:

*‘I used to get post-inflammatory hyperpigmentation very, very badly. And I think that is what led to me being very self-conscious about things like going out or taking photos … maybe it doesn’t look as bad, but to me all I can see is that hyperpigmentation which I think looks horrible.’* (P11, 22 years, Asian male, eczema)

*‘Over the years, as my skin tone has got darker, I struggled with that. And I didn’t want to be a darker shade of me.’* (P3, 43 years, Asian female, eczema)

*‘I think it’s more of a form of body dysmorphia in a way.* [The hyperpigmentation] *might not be bad to other people because family and friends will say, “oh you’re skin’s fine”. But for me, the way it makes me feel, I don’t even want to go out, I don’t want to go to places, I don’t want to meet people.’* (P13, 46 years, Black male, acne)

Several participants discussed hyperpigmentation in the context of colourism and skin-lightening practices, with one Asian female explaining that *‘lighter skin is the goal’* and *‘if you have eczema you’re just moving away from that goal’*. An Asian male was encouraged to bleach his skin as a child and was taken to a priest because he was thought to be *‘cursed’*, demonstrating the problems that can arise from cultural misunderstanding and *‘not being able to talk about hyperpigmentation with anyone’*.

Despite its profound impact, most said dyspigmentation was not discussed at medical appointments and some felt *‘embarrassed or ashamed’* to discuss it. When it was discussed, some experienced *‘dismissive language’* and were *‘made to feel like it’s really trivial’*. Some felt the aim in appointments is *‘to clear the eczema’* so hyperpigmentation is *‘not really focused on’*, and *‘you shouldn’t be complaining because your eczema is clear’*. One Asian female was made to feel *‘at fault’* by her healthcare professional because venepuncture was more challenging in hyperpigmented areas.

Many were concerned about the time taken for hyperpigmentation to resolve and limited treatment options. Most were not provided with specific treatment advice by clinicians and were told *‘it will go when your skin’s controlled’*. One participant felt a discussion around the cause, duration, and treatment for hyperpigmentation would have been helpful, whereas another felt if clinicians had *‘noticed’* and *‘validated’* her hyperpigmentation, *‘that probably would have just been enough’*.

### Challenges with structural racism

Several individuals, mostly of a Black ethnicity, highlighted issues of structural racism, stigmatisation, and bias in the context of having an inflammatory skin condition. One participant said he *‘hated his own skin’* and felt *‘inferior’* in a society where *‘you’re already trying to survive microaggressions and systemic bias’*. He also stated:

*‘I think you just have a psychological role of being visibly different* [with psoriasis]*. You know, we live in a world that already polices Blackness. So at that point you are watched … examined, you’re just in a shop and you could turn and someone is looking at you, staring at you. People stare at your body and some won’t even sit next to you. And you just learn to shrink, to fold yourself inside, mentally and emotionally.’* (P20, 29 years, Black male, psoriasis)

An Asian participant said:

*‘There’s so much racism, but when you’ve got something that’s different and makes you stand out, you become an easier target.’* (P2, 41 years, Asian female, psoriasis)

Negative experiences were often exacerbated by societal misunderstanding regarding the aetiology of inflammatory dermatoses, particularly misconceptions about poor hygiene and infectious causes:

*‘Some people would pass me at the time and say, “oh, I don’t want to catch that skin thing” because they feel it’s something bad like a disease that could kill you because of a lack of understanding.’* (P20, 29 years, Black male, psoriasis)

One Asian female with psoriasis was asked if she had *‘monkeypox’*, and another Black female with eczema was told by her manager that she needed to maintain *‘the right hygiene practices’*. A participant said people attribute his eczema to poor hygiene as they *‘see a Black man as someone who is a bit dirty’*, causing him to cover up in public.

Some felt *‘dismissed’* and discriminated against by clinicians because of their skin tone and ethnicity. One Black male said clinicians were *‘confidently and comfortably attending other White folks’* but he noticed they were *‘withdrawn’* during his own consultations, which he attributed to racism. Another felt she had been *‘treated a bit different’* owing to a *‘lack of understanding’* about her skin colour, and one individual felt her GP was *‘disparaging’* because she was a *‘minority ethnic woman’*.

There were also several concerns raised by Black participants regarding challenges accessing health care. One individual explained *‘there’s a deep failure with people of colour accessing healthcare services’* and *‘the system is against you’*. Another suggested that clinicians could *‘reach out to neighbourhoods and attend to people’* with dermatological issues who *‘cannot find their way to a healthcare centre or cannot really afford it’*.

## Discussion

### Summary

This UK-based qualitative study is the first, to the authors’ knowledge, to explore the experiences of living with an inflammatory dermatosis in people with skin of colour. Care for these individuals necessitates important ethnic and cultural considerations. The qualitative themes generated have been condensed into seven practical points for primary care clinicians to consider in everyday practice and are a step towards facilitating mutual understanding and improving care for people with skin of colour (Box 1).

**Box 1.** Practical points for primary care

Practical point	Explanation
Educate yourself and others to avoid delayed diagnosis	Unfortunately, a delayed or missed diagnosis of inflammatory skin disease is still affecting people with skin of colour in primary care Reflect on and proactively seek out opportunities to develop your knowledge on the clinical presentation of inflammatory dermatoses in people with skin of colour to help prevent further delayed diagnoses
Consider the impact of your own ethnic background on the consultation	Be mindful of how your own ethnicity and knowledge base may influence the effectiveness of a consultation with someone with skin of colour Some patients may have a strong preference for clinicians of the same ethnicity, whereas for others culturally sensitive care and effective communication are more important
Assess for and be prepared to discuss dyspigmentation with your patients	Many patients want more information on dyspigmentation Clinicians should explain to patients that: dyspigmentation is usually a result of skin inflammation so controlling the underlying condition is important; it normally fades over time, although this may take months to years; and it is exacerbated by sun exposure so photoprotection is crucial It is also important to ask about and acknowledge the psychosocial impact dyspigmentation may have on patients
Complementary treatment use may be more common in certain ethnic groups so always ask about them	Clinicians should always ask about complementary medicine use, particularly when seeing patients from diverse ethnic backgrounds, as it may have adverse effects and interfere with effective medical treatment Use may be linked to traditional cultural beliefs about health and encouraged by friends and family
Be aware of cultural misconceptions about skin disease aetiology and treatment, and do not assume patient knowledge	In some ethnic groups there is still misunderstanding, misinformation, and a lack of dialogue surrounding inflammatory dermatoses that may potentiate stigmatisation, psychological distress, and delayed presentation Do not assume patient knowledge — clarifying understanding may help improve patient satisfaction and treatment adherence
Direct your patients to reliable information sources that include diverse images and information specific to people with skin of colour	Direct people with skin of colour to resources relevant to their skin tone and ethnicity Examples include the British Association of Dermatologists (https://www.skinhealthinfo.org.uk/support-resources/skin-of-colour/), Skin of Colour Training UK (https://www.soctuk.org), and Black Skin Directory (https://www.blackskindirectory.com), which provide useful online resources for both patients and clinicians Online image banks such as Skin Deep (https://dftbskindeep.com) and Global Skin Atlas (https://www.globalskinatlas.com ) also provide free high-quality photographs of conditions in diverse skin tones
When discussing participation in research, be mindful of concerns people with skin of colour may have about taking part and dispel mistaken beliefs	Where opportunities arise, identify and dispel mistaken beliefs about research and encourage people with skin of colour to take part Diverse representation improves the generalisability of findings and will enhance future patient confidence in and adherence to dermatological treatments

### Strengths and limitations

Study strengths include its focus on an underexplored research area and representation of experiences from across the UK and from a range of ethnic groups. The qualitative study design allowed in-depth exploration of personal experiences and was the most appropriate choice for this research topic.

Limitations include the online recruitment and data collection that excluded non-English language speakers and those who do not use social media. We relied on patient-reported diagnoses and ethnicity, and three participants declined to have their camera on during the interview. Individuals with acne, male participants, and older adults were underrepresented so their experiences may be less well captured.

The first author conducted all interviews and is a practising dermatologist who identifies as a White British female. Racial and ethnic discordance has been reported to affect communication in both the clinical and research setting, particularly among those of Black ethnicity.^27,28^ Britton suggests this discordance can both facilitate and obstruct qualitative research, and recommends a reflexive awareness of it.^29^ The first author’s clinical role may have influenced data analysis, although double coding and meetings to review emerging findings with the senior author and the second author ensured a diversity of perspectives.

### Comparison with existing literature

Unfortunately, a delayed or missed diagnosis in primary care is still affecting people with skin of colour. This may partly be explained by their underrepresentation in educational resources and medical curriculae,^30^ translating to lower clinician confidence.^31^ Poor hygiene, infection, and infestation are frequent misdiagnoses that potentially exacerbate stigma surrounding inflammatory dermatoses in some ethnic groups. This could be attributed to the disproportionate representation of dark skin illustrating stigmatising infectious diseases in medical textbooks.^32^ Additionally, most outcome measures were predominantly developed in White patients.^33^ Adaptations to existing tools, such as using a *‘grey scale’* in place of erythema, have been suggested but are yet to be validated and widely accepted in clinical practice.^34^

Inflammatory dermatoses carry a significant psychosocial burden, which may be greater in certain ethnic groups.^35^ In particular, dyspigmentation can have a profound impact on people with skin of colour, with sequelae including body dysmorphia, covering up, and social isolation.^36^ It is also important to consider this in the context of colourism, skin-bleaching practices, and spiritual beliefs, a concept not previously explored in the literature, to the authors’ knowledge. Regarding its aetiology, dyspigmentation is typically a consequence of skin inflammation.^37^ However, high-potency topical steroids can cause hypopigmentation in darker skin types, which may affect treatment adherence.^38^

The use of complementary medicines for inflammatory dermatoses is well documented across all skin tones,^39^ but has been linked with ethnicity and cultural beliefs.^40,41^ The decision to use complementary treatments may be related to traditional beliefs about health, illness, and remedies,^41^ and encouraged by friends and family.^40^ Their use may have adverse effects and delay effective medical treatment.^42^

Misunderstanding, misinformation, and a lack of dialogue surrounding inflammatory dermatoses remain a challenge in certain ethnic groups and may potentiate stigmatisation, psychological distress, and delayed presentation. This may be exacerbated by the lack of diverse images of inflammatory dermatoses online.^43^ Social media platforms have therefore become an information source for many patients, but these are often unregulated and led by people who lack medical qualifications, which may exacerbate misinformation.^44^

Some participants had a strong preference for clinicians of the same ethnicity whereas others prioritised knowledge and communication skills. A recent study from the US found that Black patients were more likely than other ethnic groups to prefer a race-concordant dermatologist, although cultural understanding was deemed to be more important.^45^ The impact of structural racism, stigmatisation, and bias on people with skin of colour should also be considered, as these factors may intensify the psychosocial impact of having an inflammatory skin condition.^46^

### Implications for research and practice

The themes generated have been condensed into seven practical points for primary care clinicians to consider and integrate into everyday practice. These are listed here, and expanded with examples in Box 1:

- educate yourself and others to avoid delayed diagnosis;
- consider the impact of your own ethnic background on the consultation;
- assess for and be prepared to discuss dyspigmentation with your patients;
- complementary treatment use may be more common in certain ethnic groups so always ask about them;
- be aware of cultural misconceptions about skin disease aetiology and treatment, and do not assume patient knowledge;
- direct your patients to reliable information sources that include diverse images and information specific to people with skin of colour; and
- when discussing participation in research, be mindful of concerns people with skin of colour may have about taking part and dispel mistaken beliefs.

Diversification of medical curricula and learning resources is essential to support a timely diagnosis for patients with skin of colour. Skin assessment tools and outcome measures also need to be adapted and validated for use in darker skin tones. To address misinformation among certain groups, future research could focus on culturally tailored community engagement and outreach programmes to raise awareness and promote early treatment. Underrepresentation of people from diverse ethnic groups in research is multifactorial but research mistrust is an important barrier. Diversifying representation within research teams and engagement with community partners during study recruitment could help build trust and encourage participation.

In conclusion, these findings offer insight into the complex experiences and challenges faced by UK adults with skin of colour living with eczema, acne, and psoriasis. We hope the results encourage open dialogue between primary care clinicians and patients to foster mutual understanding, identify challenges, and enhance patients’ experiences, which is a meaningful step towards positive change.

## Data

The data underlying this article cannot be shared publicly for ethical/privacy reasons. Representative quotes are incorporated into the article.
